# Overexpression of mitochondrial creatine kinase preserves cardiac energetics without ameliorating murine chronic heart failure

**DOI:** 10.1007/s00395-020-0777-3

**Published:** 2020-01-10

**Authors:** Fang Cao, Mahon L. Maguire, Debra J. McAndrew, Hannah A. Lake, Stefan Neubauer, Sevasti Zervou, Jürgen E. Schneider, Craig A. Lygate

**Affiliations:** 1 Division of Cardiovascular Medicine, Radcliffe Department of Medicine, University of Oxford, Roosevelt Drive, Oxford, OX3 7BN UK; 2 The BHF Centre for Research Excellence, Oxford and The Wellcome Centre for Human Genetics, University of Oxford, Roosevelt Drive, Oxford, OX3 7BN UK; 3 Present Address: Centre for Preclinical Imaging, Sherrington Building, Crown Street, Liverpool, UK; 4 Present Address: Experimental and Preclinical Imaging Centre (ePIC), Leeds Institute of Cardiovascular and Metabolic Medicine, University of Leeds, Leeds, UK

**Keywords:** Cardiac energetics, Metabolism, Creatine kinase, Heart failure, Transgenic

## Abstract

Mitochondrial creatine kinase (Mt-CK) is a major determinant of cardiac energetic status and is down-regulated in chronic heart failure, which may contribute to disease progression. We hypothesised that cardiomyocyte-specific overexpression of Mt-CK would mitigate against these changes and thereby preserve cardiac function. Male Mt-CK overexpressing mice (OE) and WT littermates were subjected to transverse aortic constriction (TAC) or sham surgery and assessed by echocardiography at 0, 3 and 6 weeks alongside a final LV haemodynamic assessment. Regardless of genotype, TAC mice developed progressive LV hypertrophy, dilatation and contractile dysfunction commensurate with pressure overload-induced chronic heart failure. There was a trend for improved survival in OE-TAC mice (90% vs 73%, *P* = 0.08), however, OE-TAC mice exhibited greater LV dilatation compared to WT and no functional parameters were significantly different under baseline conditions or during dobutamine stress test. CK activity was 37% higher in OE-sham versus WT-sham hearts and reduced in both TAC groups, but was maintained above normal values in the OE-TAC hearts. A separate cohort of mice received in vivo cardiac ^31^P-MRS to measure high-energy phosphates. There was no difference in the ratio of phosphocreatine-to-ATP in the sham mice, however, PCr/ATP was reduced in WT-TAC but preserved in OE-TAC (1.04 ± 0.10 vs 2.04 ± 0.22; *P* = 0.007). In conclusion, overexpression of Mt-CK activity prevented the changes in cardiac energetics that are considered hallmarks of a failing heart. This had a positive effect on early survival but was not associated with improved LV remodelling or function during the development of chronic heart failure.

**Electronic supplementary material:**

The online version of this article (10.1007/s00395-020-0777-3) contains supplementary material, which is available to authorized users.

## Introduction

Effective treatments for chronic heart failure often act to reduce cardiac energy demand by lowering afterload and/or heart rate (e.g. β-adrenergic antagonists, ACE inhibitors and diuretics). However, this approach is limited by the risk of bradycardia and hypotension, and an attractive alternative for new heart failure therapies is therefore to also improve myocardial energy supply [12, 34].

The creatine kinase (CK) phosphagen system has a central role in matching energy supply to energy demand and represents a potential target for therapeutic intervention. The mitochondrial isoform of CK (Mt-CK) is located within the mitochondrial intermembrane space, where it is functionally coupled to ATP production via the adenine nucleotide translocase (ANT). Mt-CK catalyses the transfer of a phosphoryl group from the newly generated ATP onto creatine to form phosphocreatine (PCr) and ADP (which stimulates further oxidative phosphorylation) [40]. PCr is relatively smaller and less polar than ATP allowing it to accumulate to higher levels and act as an energy transport and buffering system, capable of regenerating ATP when demand outstrips supply. This reaction is catalysed by cytosolic CK isoforms, such as muscle (M-CK), that are often functionally coupled to major ATP-using enzymes (e.g. myosin ATPase, SERCA) [40].

A substantial body of observational data in patients and animal models has implicated an impaired CK system in the pathophysiology of chronic heart failure (CHF) [15, 22], with both total creatine levels and CK activity reduced commensurate with disease severity (reviewed in [34]). This is most commonly observed in vivo as a fall in the PCr/ATP ratio using ^31^phosphorus-magnetic resonance spectroscopy (^31^P-MRS). For example, in patients with dilated cardiomyopathy, a low PCr/ATP was associated with higher mortality and correlated with New York Heart Association class and ejection fraction [35, 36].

However, whether loss of CK function has a causative role in the pathophysiology of CHF has yet to be proven. Genetic knockout studies in mice have suggested only mild or ambiguous effects on cardiac function, suggesting that a dysfunctional CK system, in itself, is usually insufficient to cause heart failure [5, 23, 24]. Even when loss of function has been superimposed on surgical models of CHF, the expected detrimental effects have not been obvious [13, 20, 32].

A complementary approach is to ask whether preventing energetic remodelling via augmentation of the CK system would be beneficial. Increasing myocardial levels of creatine protected against ischaemia–reperfusion injury (I/R), but did not improve outcomes in the setting of CHF [6, 21]. However, seminal evidence comes from overexpression of M-CK in mouse heart, which improved survival and contractile function in a model of pressure overload-induced CHF. Most convincingly, this was associated with preservation of ATP production via the CK reaction (CK flux), and protection was lost in a sub-group of mice where the transgene was turned off partway through the protocol [9].

The therapeutic potential for augmentation of Mt-CK in CHF has yet to be tested and, quite apart from it being a major determinant of PCr/ATP [49], there are good reasons to think that this approach may also be beneficial. Not least, is an effect to reduce opening probability of the mitochondrial permeability transition pore (mPTP), thereby preventing cardiomyocyte death [48, 50]. This is a key event governing I/R injury where Mt-CK overexpression has already been shown to be cardioprotective [48], and it is also thought to contribute to cellular loss in the chronically failing heart. Preserving mitochondrial health is an important goal in heart failure, and the close functional coupling between Mt-CK and oxidative phosphorylation is thought to promote this. A further contributory factor is likely the role of Mt-CK in promoting mitochondrial structural integrity by forming contact sites between the inner and outer membranes [40]. Finally, Mt-CK is particularly vulnerable to deactivation via reactive oxygen species [47], which are known to be elevated in the failing heart. Replacement of affected protein could prove beneficial, especially since Mt-CK activity closely correlates with recovery of LV function in post-ischaemic myocardium [2].

For the reasons outlined above, we hypothesised that overexpression of Mt-CK would be beneficial in terms of cardiac energetics, function and structural remodelling in a mouse model of pressure overload CHF. We utilised an existing strain of cardiac-specific Mt-CK-OE under control of the α-MHC promoter that was generated in our laboratory [48]. This used a cautious transgenic approach to increase myocardial Mt-CK activity by 25–30%, which had no measureable effect on baseline mitochondrial function, but was sufficient to provide protection against I/R injury.

## Methods

### Ethics statement and colony maintenance

All animal experiments were approved by the Committee for Animal Care and Ethical Review at the University of Oxford, and comply with Home Office Regulations incorporating the Animals (Scientific Procedures) Act of 1986 and Directive 2010/63/EU of the European Parliament (project licence number 30/3314). Mice were maintained in individually ventilated cages on a 12 h night/day cycle with controlled temperature (21 °C) and humidity. Mice were fed irradiated Global Diet 2916 (Envigo, Huntingdon, UK) and water ad libitum and housed with littermates in specific pathogen-free conditions. Creation of the cardiac-specific transgenic CK-Mt-OE line and genotyping protocol has been described previously [48]. This strain has been backcrossed for > 10 generations using C57BL/6JOlaHsd mice obtained from Envigo, Huntingdon, UK (formerly known as Harlan). For all experiments, transgenic mice were homozygote for CK-Mt overexpression and wild-type (WT) were non-transgenic littermates bred in our establishment.

### Study design

Male WT and overexpressing (OE) mice aged 15 ± 3 weeks were randomly assigned to either transverse aortic constriction (TAC) or sham surgery to create four experimental groups: WT-Sham, OE-Sham, WT-TAC, and OE-TAC. All mice had an echocardiogram a few days before surgery (week 0) and at week 3 and week 6 post-surgery to assess onset of chronic heart failure. Left ventricular (LV) haemodynamics were assessed 2–3 days after the final echocardiogram, after which animals were killed by removal of the heart under terminal anaesthesia. LV tissue was washed briefly in physiological saline, blotted and weighed, then snap frozen using Wollenberger tongs in liquid nitrogen and stored at − 80 °C until use. An independent cohort of mice received TAC or sham surgery and were subjected to ^31^P-MRS for analysis of in vivo PCr/ATP ratio at 6 weeks post-surgery. Supplementary Table 1 shows animal numbers and fate for all experimental steps.

### Surgery

TAC surgery was performed aseptically as described previously under isoflurane general anaesthesia (2% in medical oxygen with anaesthetic depth assessed by loss of pedal reflex) [18, 27]. Buprenorphine analgesia was given subcutaneously immediately prior to surgery (0.8 mg/Kg) and again the morning after surgery (0.4 mg/Kg).

### Echocardiography and haemodynamics

Mice were maintained on isoflurane anaesthesia (1.0–1.5% in medical O_2_), placed on a homeothermic table and parasternal short-axis B-mode views obtained at the level of the papillary muscles using a Visualsonics Vevo 2100 ultrasound system with 22–55 MHz transducer (MS 550D). Data analysis was performed by a single operator blinded to genotype using Vevo Lab, Edition 1.7.1 (Visualsonics, Toronto, Canada). Measurements were made directly from the 2-D images and are therefore expressed as areas.

Retrograde LV cannulation was performed under isoflurane anaesthesia via the right carotid artery using a 1.4F solid-state pressure catheter (SPR-839, Millar Instruments, Texas, USA) as described previously [5]. The jugular vein was cannulated using flame-stretch polyethylene tubing (Portex 0.96 mm OD, 800/100/200, Smiths Medical, UK) for administration of dobutamine hydrochloride at 16 ng/g BWt/min. Mice were killed at the end of the experiment by overdose of pentobarbitone.

### Biochemistry

Frozen LV tissue was powdered and total creatine (i.e. the sum of free creatine and phosphocreatine (PCr)) was quantified by HPLC [43]. Total creatine kinase (CK) and citrate synthase activities were quantified under saturating conditions using standard spectrophotometric techniques and relative CK isoenzyme activities via the SAS-1 gel electrophoresis system (Helena Biosciences, UK). Values were normalised to protein content measured by the Lowry method, as described previously [48]. The relative maximal velocity of the CK reaction was estimated by the product of enzyme activity and total creatine concentration, since these two parameters represent the numerators of the rate equation that vary the most under disease conditions and are therefore the primary determinants [14].

### In vivo ^31^P-MRS

General anaesthesia was maintained using 1.5–2.0% isoflurane in oxygen with mice placed prone in a heated cradle with the heart directly over the surface coil array and ECG and respiratory gating [4]. All ^31^P-MRS data were acquired utilising a horizontal 9.4 T magnetic resonance system equipped with a Direct Drive2 console and 120 mm i.d., 0.6 T/m, shielded gradient set (Agilent Technologies, USA) operating at 400 MHz frequency for ^1^H and 168 MHz for ^31^P measurements. Images were acquired using a linear double-tuned, actively decoupled, ^1^H/^31^P 39 mm birdcage resonator (i.d. 39 mm) and a 14 mm actively decoupled quadrature surface coil array for ^31^P signal reception (Rapid Biomedical, Germany). Anatomical scout images were acquired using the ^1^H channel of the birdcage resonator for both transmit and receive.

Two-dimensional, density-weighted, ^31^P chemical shift imaging (2D-CSI) was used in short-axis orientation to acquire spectroscopic measurements in vivo (FOV 30 × 30 mm^2^, 16 × 16 PE steps, 5 mm slice thickness, threefold undersampled, 2600 FIDs, 60° flip angle, cardiac triggered, TR ≈ 250 ms [i.e. two cardiac cycles], total acquisition time ~ 25 min); the resulting spatial resolution was approximately 1.9 × 1.9 × 5.0 mm (voxel volume of 17.6 μL). Prior to Fourier transform, the data were zero-filled to 64 × 64 PE steps to improve the apparent spatial resolution of the images, and a line broadening of 60 Hz was applied to improve the SNR of the resulting spectra.

The data were reconstructed using IDL 8.2 (Harris Geospatial Solutions, USA) and spectra corresponding to voxels placed in the myocardium and blood were fitted in the time domain using in-house software [28]. Correction for myocardial signal contamination from blood, and T1 saturation effects was carried out in Excel 2014 (Microsoft Corporation, USA); T1 values for PCr and ATP in the mouse myocardium at 9.4 T were taken from the literature [7].

### Analysis and statistics

Researchers were blinded to experimental groups for all data analysis. A log-rank (Mantel–Cox) test was used to determine whether Kaplan–Meier survival curves were significantly different. Echocardiography data were analysed by two-way repeated-measures ANOVA with Tukey’s multiple comparison test. Haemodynamic, organ weights and biochemistry data were checked for normality using a D’Agostino–Pearson (K2) test and analysed by one-way ANOVA with Sidak’s multiple comparison test if normally distributed, or, by nonparametric Kruskal–Wallis test with Dunn’s correction if not. Four comparisons were predetermined to be made for all analyses: WT-Sham vs. OE-Sham; WT-Sham vs. WT-TAC; OE-Sham vs. OE-TAC; WT-TAC vs. OE-TAC.

## Results

### Survival

**Fig. 1:**
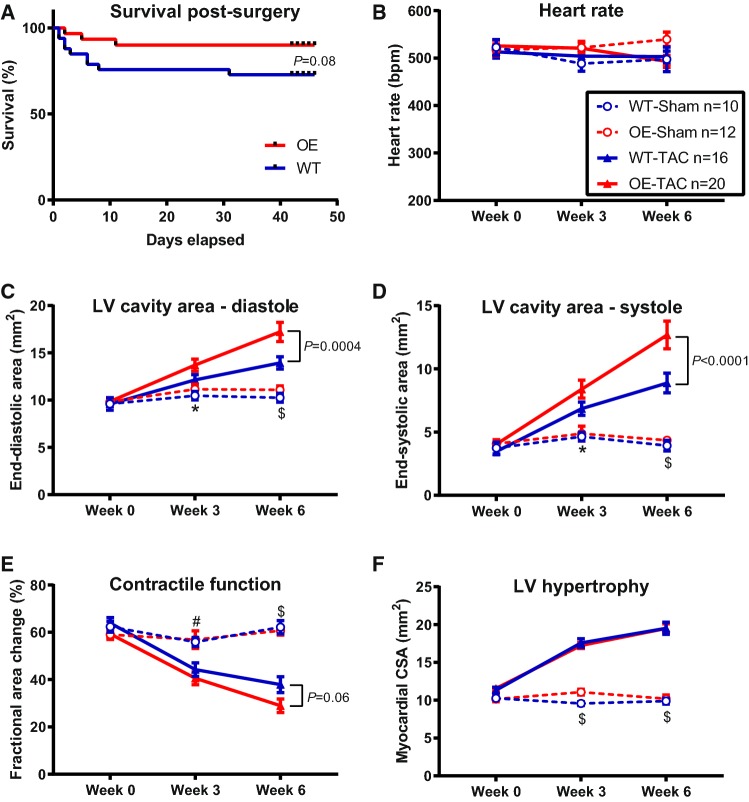
Survival and echocardiographic parameters in sham and transverse aortic constriction (TAC) operated mice. **a** Kaplan–Meier survival curve for wild-type (WT, *n* = 33) and Mt-CK overexpressing (OE, *n* = 30) mice following TAC surgery for the main study and MRS sub-study combined. Sham groups are not shown since there were no procedural deaths. **b***–***f** Echocardiography parameters derived from 2-D parasternal short-axis views and therefore expressed as areas. The legend and numbers given in (**b**) apply to all panels: WT-Sham n = 10, OE-Sham *n* = 12, WT-TAC *n* = 16; OE-TAC *n* = 20. There were no significant differences between sham groups for any parameter at any time point. Both TAC groups showed a progressive LV dilation compared to shams during diastole (**c**) and systole (**d**) and was significantly more pronounced in the OE mice. **e** This manifested as a greatly reduced contractile function in both TAC groups compared to sham controls, but to a broadly similar extent. **f** shows myocardial cross-sectional area as an indicator of LV hypertrophy, which was highly significant in both TAC group irrespective of genotype. Data shown are mean ± standard error with analysis by two-way repeated measures ANOVA with Tukey’s multiple comparison test, *P < 0.05 for OE-Sham versus OE-TAC; ^#^*P* < 0.05 and ^$^*P* < 0.001 for both sham groups versus their respective controls

The total number of animals entering the study and their subsequent fate are detailed in Supplementary Table 1. The Kaplan–Meier survival curves for the TAC surgery groups (Fig. 1) show a strong trend for improved survival in the OE mice compared to WT (3 deaths from *n* = 30 OE mice vs. 9 deaths from *n* = 33 WT, *P* = 0.08), in particular, within the first 10 days post-surgery. An analysis of the causes of death (Supplementary Table 2) indicates that WT-TAC mice had more incidence of acute and chronic heart failure during that period. Deaths during haemodynamic examination were spontaneous rather than due to surgical error and affected more in the OE group.

### LV remodelling and function

**Fig. 2:**
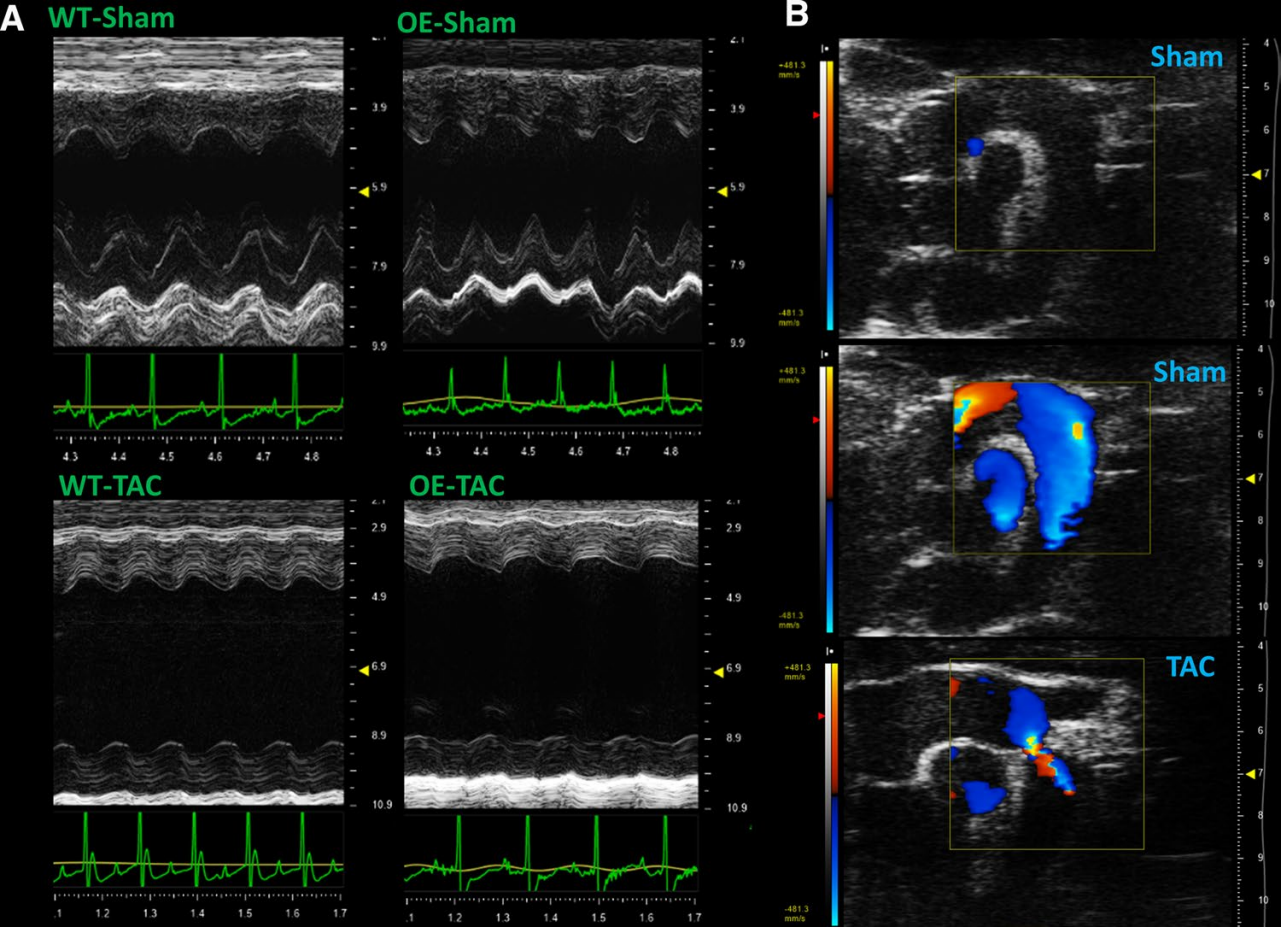
Representative echocardiograms obtained 6 weeks after transverse aortic constriction (TAC) or sham surgery. **a** Shows M-mode images from the parasternal short-axis view from all four experimental groups, representing wild-type (WT) and Mt-CK overexpressing mice (OE). **b** Shows sagittal views through the aortic arch with the aortic root in the upper left of each image. The upper and middle images are from a sham-operated mouse shown with and without colour Doppler for anatomical reference. The lower image illustrates the severe aortic stenosis that was observed in all TAC operated mice

**Fig. 3:**
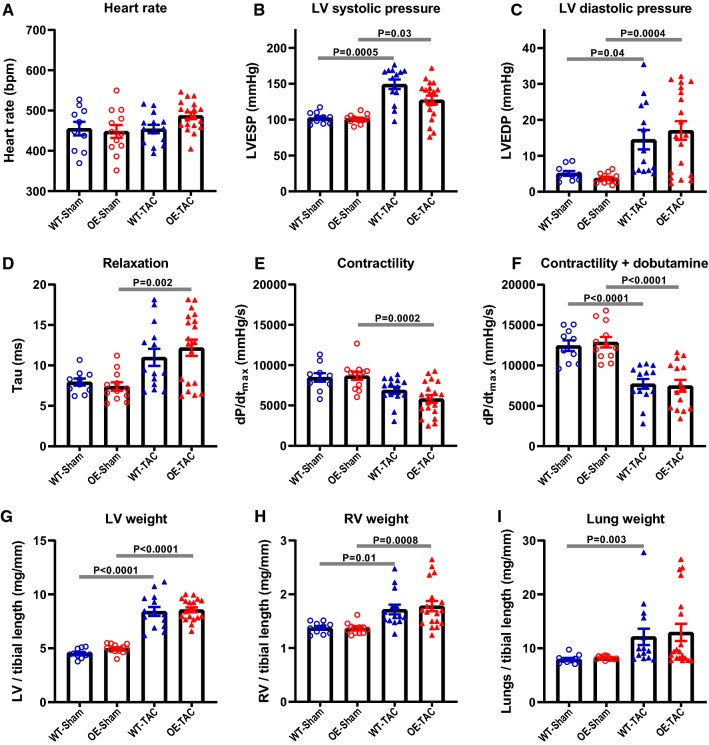
Left ventricular haemodynamics and organ weights obtained 6 weeks after sham or transverse aortic constriction (TAC). **a** Heart rate, **b** LV end-systolic pressure, **c** LV end-diastolic pressure, **d** isovolumetric constant of relaxation, tau, as a measure of relaxation, **e** the rate of pressure rise maximum (dP/dt_max_) as a measure of contractility, **f** dP/dt_max_ during IV infusion with dobutamine at 16 ng/g BW/min as a measure of contractile reserve. Organ weights were obtained post-mortem and normalised to tibial length for **g** left ventricle, **h** right ventricle and **i** lung weight. Directional changes for all parameters are consistent with severe LV hypertrophy and subsequent development of a heart failure phenotype in TAC animals, which was not affected by genetic modification. Data shown are mean ± standard error for WT-Sham *n* = 10, OE-Sham *n* = 12, WT-TAC *n* = 14; OE-TAC *n* = 19. Analysis by one-way ANOVA with Sidek’s multiple comparison test, except for data shown in panels **b**, **f**, **h**, and **i**, which failed the normality test and were analysed by Kruskal–Wallis with Dunn’s correction

No differences in echocardiographic parameters were evident between WT and Mt-CK-OE animals prior to surgery (week 0) or between sham-operated groups at any time point (Fig. 1). Both TAC groups exhibited overt LV dilatation, contractile dysfunction and LV hypertrophy compared to their sham controls (Fig. 1b–f and representative images in Fig. 2). By 6 weeks post-surgery, the hearts from OE-TAC mice were significantly more dilated than WT-TAC, with a trend towards impaired contractility (Fig. 1e), but with no differences in hypertrophic response (Fig. 1f and confirmed at post-mortem Fig. 3g).

A similar pattern was observed for the LV haemodynamic data. There were no significant differences between WT and OE-sham groups, while both TAC groups had significantly impaired LV function compared to their respective sham controls, indicative of chronic heart failure (Fig. 3 and Supplementary Table 3). In both TAC groups, a similar proportion of mice had signs of pulmonary congestion, as evidenced by elevated RV and lung weights at post-mortem (Fig. 3h, i). Mt-CK overexpression did not significantly alter any of these parameters.

### Cardiac energetics

Total creatine kinase activity was 37% higher in OE-sham compared to WT-sham hearts (Table 1). As expected, values were lower in both TAC groups, but only in OE hearts was total CK activity maintained above normal WT levels despite the development of heart failure. In OE-sham hearts the relative content of MM-CK isoforms was paradoxically higher, but this was normalised in the OE-TAC hearts, where higher Mt-CK activity made the largest contribution to maintaining total CK activity. No differences in total creatine levels or citrate synthase activity were detected between any groups.

**Table 1:** LV myocardial enzyme activities and creatine levels

	WT-Sham (*n* = 10)	OE-Sham (*n* = 11)	WT-TAC (*n* = 17)	OE-TAC (*n* = 20)
Total CK (IU/mg protein)	6.36 ± 0.42	8.74 ± 0.64**	6.14 ± 0.36	6.85 ± 0.37^#^
Mt-CK (IU/mg protein)	1.60 ± 0.20	2.02 ± 0.20	1.14 ± 0.11	1.58 ± 0.09^§^
MM-CK (IU/mg protein)	4.36 ± 0.43	6.15 ± 0.49*	4.69 ± 0.29	4.93 ± 0.36
MB-CK (IU/mg protein)	0.36 ± 0.07	0.55 ± 0.13	0.23 ± 0.02	0.28 ± 0.02^#^
BB-CK (IU/mg protein)	0.03 ± 0.01	0.02 ± 0.01	0.08 ± 0.02	0.06 ± 0.01
Citrate synthase (IU/mg protein)	0.75 ± 0.05	0.81 ± 0.07	0.69 ± 0.05	0.66 ± 0.06
Total creatine (nmol/mg protein)	59 ± 2	66 ± 3	56 ± 3	58 ± 3

Data are mean ± SEM and expressed per milligram of protein. *P < 0.05 and **P < 0.01 for WT-Sham vs OE-Sham; ^#^*P* < 0.05 for OE-Sham vs OE-TAC; ^§^*P* < 0.05 WT-TAC vs OE-TAC by one-way ANOVA and Sidak’s multiple comparison test

**Fig. 4:**
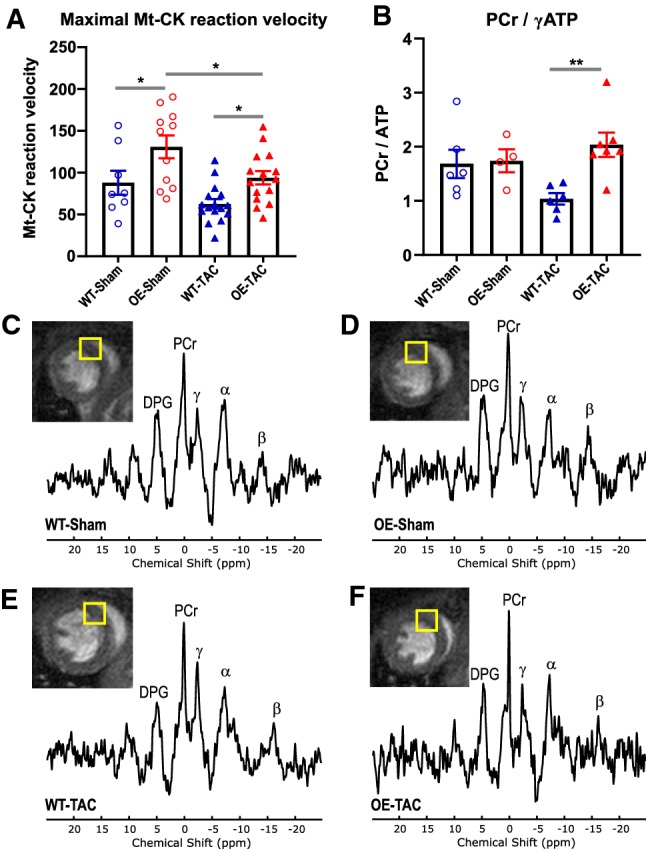
Cardiac energetics 6 weeks after sham or transverse aortic constriction (TAC). **a** Estimate of maximal Mt-CK reaction velocity calculated as product of Mt-CK activity and creatine concentration in ex vivo LV tissue. Mean ± standard error from WT-Sham *n* = 8, OE-Sham *n* = 11, WT-TAC *n* = 16; OE-TAC *n* = 15, with one-way ANOVA and Sidek’s multiple comparison test *P = 0.03. **b** Ratio of phosphocreatine (PCr) to γATP peak measured in vivo by ^31^P-MRS. Mean ± standard error from WT-Sham n = 6, OE-Sham *n* = 4, WT-TAC *n* = 6; OE-TAC *n* = 7, with one-way ANOVA and Dunn's correction, **P = 0.007. **c***–***f** Representative myocardial spectra from each of the four experimental groups showing peaks for 2,3-Diphosphoglycerate (DPG), PCr and the *γ*, *α*, and *β* phosphoryl groups of ATP alongside corresponding short-axis scout image

To provide a more sensitive indicator of CK activity, an estimate of relative reaction velocity through Mt-CK was calculated by multiplying by the total creatine concentration (as per [45]). This confirmed that Mt-CK reaction velocity was elevated in OE-sham hearts and was lower in TAC failing hearts, but was nevertheless maintained at supra-normal levels in OE-TAC (Fig. 4).

The ratio of phosphocreatine to ATP was measured in vivo in a separate cohort of mice and representative scouting image and spectra are shown in Fig. 4. There was no difference in PCr/ATP between sham groups, but the clear trend for lower PCr/ATP in WT-TAC hearts was completely prevented by Mt-CK-OE, such that PCr/ATP was significantly higher in OE-TAC versus WT-TAC hearts (*P* = 0.007) thereby maintaining normal baseline levels (Fig. 4b).

## Discussion

Here we demonstrate for the first time that overexpression of the mitochondrial isoform of CK preserves normal levels of Mt-CK activity and PCr/ATP ratio in the chronically failing mouse heart. The consequences proved nuanced, since although more Mt-CK-OE mice survived the acute compensatory stage following TAC surgery, they were not subsequently protected from adverse LV remodelling or contractile dysfunction measured at later time points.

There was a clear trend towards improved early survival in the Mt-CK-OE mice (90% vs. 73% for WT), which can be attributed to a higher incidence of heart failure in WT during the first 10 days. This did not quite reach statistical significance, in part, because mortality in the WT group was relatively low (cf. [9]), which inevitably limits the potential effect size and therefore the power of the study to detect it. It is therefore possible and plausible that OE mice are partially protected during the early period of adaptation to acute pressure overload, since workload, and therefore energetic requirements, are high while compensatory adaptations are still developing. This would not be detected by our in vivo assessments at 3 and 6 weeks and therefore further studies of this early response are merited.

We cannot rule out that survival bias may have influenced the later time points. For instance, if mice survived that would otherwise have died during the acute phase, then we would expect them to show more severe indices of heart failure. This could explain why Mt-CK-OE hearts exhibited greater LV dilatation and were more likely to spontaneously stop beating during the stress of haemodynamic assessment.

In all chronic heart failure studies of this type, these is a trade-off between animal welfare and the length of follow-up. It could be argued that the extent of heart failure was not severe enough or sufficiently prolonged, since we did not observe high mortality or large falls in total CK activity or in myocardial creatine levels [Cr]. In this context, was the energetic deficit too mild to demonstrate a rescue? One consideration is the presence of a missense mutation in nicotinamide nucleotide transhydrogenase (*Nnt*) in the C57BL/6J strain, which has been shown to reduce oxidative stress and ameliorate heart failure induced by TAC [37]. However, this is not a factor here, since our transgenic mice and controls were created from the C57BL/6JOlaHsd substrain, which does not harbour the *Nnt* mutation [8]. Our previous experience of the TAC model has shown that CK system changes only occur in the most severely affected mice, i.e. those with evidence of pulmonary congestion [22]. Even then we only observed a relatively modest 11% fall in total [Cr], which appears to reflect the lower starting values in this species. In the current study we observe a sizeable subset of animals with elevated lung weights suggesting pulmonary congestion and elevated RV weights indicating prolonged elevation of LV diastolic pressures. Indeed, the values for haemodynamic and echocardiographic parameters are comparable to our previous findings suggesting that we do have an effective heart failure model [22]. It is probably fair to say that ex vivo enzyme activities and substrate pools are the least sensitive measures of energetic status [25].

For this reason, we also estimated maximal reaction velocity by multiplying CK activity by total [Cr], which takes into account changes in both enzyme and substrate, since these are the major determinants of the rate equation [14, 45]. The expected drop in reaction velocity following TAC was observed in both genotypes, but absolute values remained supra-physiological in Mt-CK-OE hearts. In an independent experiment, we also performed in vivo ^31^P-MRS in these transgenic mice for the first time and showed that PCr/ATP ratio was not altered in the sham animals, but that Mt-CK-OE completely prevented the fall in PCr/ATP commonly observed in the failing heart. The absolute values obtained and the size of PCr/ATP reduction is comparable to other published values for pressure overload in human and mouse [10, 11, 29, 30].

A key question arising from this study is why improved energetics did not translate into improved cardiac function? One possibility is that overexpression of Mt-CK-OE had unknown confounding effects on mitochondria that an improvement in energetics could not overcome. Although we cannot rule this out, it seems unlikely, since we have previously shown that Mt-CK-OE does not affect mitochondrial cell density, citrate synthase activity, or mitochondrial respiration. Metabolomics indicated normal cellular metabolism and expression of closely associated proteins, ANT, VDAC, and BCL-2 were not significantly altered [48]. However, since Mt-CK-OE inhibits mPTP opening, our data raise the possibility that prolonged inhibition might be deleterious in the TAC model.

Similarly, Mt-CK could have increased reactive oxygen species (ROS) or provided a target for oxidative damage that added to the cumulative burden. It is a limitation of our study that we did not quantify the effects of Mt-CK-OE on oxidative stress, however, we have previously found no difference in mitochondrial proton leak and uncoupling, which suggests the major source of cellular ROS is unlikely to be different [48]. Mt-CK has also been found to reduce (rather than increase) ROS formation under stress conditions [31]. Furthermore, Mt-CK protein is very sensitive to oxidative damage and this is reflected in reduced Mt-CK activity [42], yet in the current study, the Mt-CK activity compared to sham was 0.46 IU/mg lower in WT compared to 0.44 IU/mg in Mt-CK-OE hearts. This suggests that mitochondrial exposure to ROS was similar in both heart failure groups.

There is no doubt that PCr/ATP is an important biomarker for the energetic status of the heart. Multiple studies across species and aetiologies have demonstrated that PCr/ATP correlates with measures of cardiac workload, e.g. ejection fraction and wall stress [16, 30, 33, 35]. Improvement in clinical condition is also often tracked by improvement in PCr/ATP [29, 33, 36].

However, PCr/ATP simply reflects relative metabolite pools and is not a specific marker. For example, the fall in this ratio is underestimated in advanced heart failure if both PCr and ATP levels are reduced [25]. PCr/ATP levels are affected by multiple conditions, often before overt contractile dysfunction is evident, e.g. in type 2 diabetes [39], hypertension [16], and obesity [38]. PCr/ATP may also change acutely, e.g. falling in response to increased workload in hearts with pre-existing disease [38].

It should, therefore, not come as a surprise that we occasionally observe a large disconnect between PCr/ATP levels and outcomes. For example, in pressure overload mice (TAC model) a maximal reduction in PCr/ATP was already observed at 3 weeks, but contractile function continued to decline and only correlated with PCr/ATP at 6 weeks [30]. Conversely, in the same TAC model by a different group, contractile dysfunction preceded the fall in PCr/ATP by 11 weeks [1]. Perhaps the most egregious example is in mice null for the GLUT4 transporter, which develop LV hypertrophy and depressed ejection fraction despite myocardial PCr/ATP that is 60% higher [46].

It is also notable that low PCr/ATP does not per se result in contractile dysfunction. Mice fed a high-fat, high-sucrose diet had a 30% reduction in PCr/ATP, but this did not adversely affect function under resting conditions [17]. Similarly, diabetic db/db mice were shown to have a low PCr/ATP early on when cardiac function was normal, but 11 weeks later, function had deteriorated at a time when PCr/ATP improved [1, 19].

It is a strength of our study that ^31^P-MRS was performed in vivo at physiological workloads, but ideally we would have liked to measure CK flux by saturation transfer, since this is considered a more sensitive indicator than PCr/ATP in chronic heart failure [3, 25]. However, the technical capability in murine heart was not available to us at the time of this study and there are very few groups worldwide that can. Nevertheless, our study shows for the first time that a metabolic intervention effective at preserving PCr/ATP is not sufficient in itself to protect the pressure-overloaded heart from developing CHF. The sole use of PCr/ATP to identify potential new therapeutic agents should therefore be approached with caution.

Our findings are in contrast to transgenic mice overexpressing the muscle isoform of CK in the heart (M-CK), which were shown to have reduced mortality and improved systolic function in the TAC model [9]. This was associated with relative preservation of PCr/ATP levels and CK flux that was maintained at control levels. The reasons for this are not immediately apparent, it is notable that protein expression of Mt-CK was found to be the main determinant of PCr/ATP and of CK flux in a porcine model of pressure overload [49]. This is supported by our own findings and we would therefore expect augmentation of Mt-CK to be a particularly attractive strategy. Perhaps the simplest explanation is one of gene dosing, the M-CK-OE model had ~ 70% increase in total CK activity, whereas it was only 37% higher in our Mt-CK-OE mouse hearts. This was a deliberate strategy on our part, since we were concerned at the potential detrimental effects of expressing too much transgenic protein within a small cellular compartment. However, while we cannot rule out that greater Mt-CK expression would bring benefits, this seems unlikely given that we successfully maintained PCr/ATP and CK reaction velocity at normal levels and this level of Mt-CK overexpression is sufficient to protect against I/R injury [48]. It is possible that the key difference relates to how well ADP is buffered at the myofilaments, since this depends on the activity of cytosolic creatine kinases (predominantly M-CK), which are typically impaired in heart failure and would not be improved by Mt-CK overexpression. ADP levels are not readily detectable by in vivo ^31^P-MRS, but experiments ex vivo have shown that elevated ADP is enough to cause diastolic dysfunction, particularly in the presence of Ca^2+^ overload [41, 44]. It may therefore be insufficient to only correct PCr/ATP and there may be synergy in increasing activity of both mitochondrial- and M- CK isoforms together.

Our findings add to the debate on whether impairment of the CK system has a causative role in heart failure progression, which we have previously reviewed in detail [26]. The fact that preventing these changes did not positively influence pathophysiology argues against causation, which is in agreement with our previous studies in several knockout models [5, 20, 23].

In conclusion, overexpression of Mt-CK in the heart successfully maintained key markers of cardiac energetics at or above normal control values. That this was insufficient to improve LV remodelling or function during the development of chronic heart failure shows that normalisation of PCr/ATP and mitochondrial creatine kinase levels are not in themselves curative. Nevertheless, we observed a trend for improved survival during the acute compensatory phase, which suggests a focus for future study.

## Electronic supplementary material

Below is the link to the electronic supplementary material.

Supplementary file1 (PDF 126 kb)
