# Supplementary material for: Overexpression of mitochondrial creatine kinase preserves cardiac energetics without ameliorating murine chronic heart failure
Source: Basic Res Cardiol. 2020 Jan 10;115(2):12. doi: 10.1007/s00395-020-0777-3 (PMC6954138; doi:10.1007/s00395-020-0777-3)
Supplement: Supplementary file 1 — Supplementary file1 (PDF 126 kb) [file 395_2020_777_MOESM1_ESM.pdf]

Supplementary material

Cao *et al.* Over-expression of mitochondrial creatine kinase preserves cardiac energetics without ameliorating murine chronic heart failure.

Main study	WT-SHAM	OE-SHAM	WT-TAC	OE-TAC
Number of mice undergoing surgery	11	13	23	21
Died / culled due to ill health	1	1	5	1
Number with complete echo data	10	12	16	20
Died during haemodynamic exam	0	0	2	1
Number with haemodynamic data	10	12	14	19
- Did not tolerate dobutamine	0	0	0	4
Excluded due to band internalisation	0	0	1	0
**NMR sub-group**				
Number of mice undergoing surgery	6	7	12	9
Died / culled due to ill health	0	1	4	2
Number with useable ^{31}P-NMR data	6	4	6	7

Supplementary Table 1 Numbers and fate of all mice entering the study. Groups were wild-type (WT) and Mt-CK overexpressing mice (OE) that received either sham-operation or transverse aortic constriction (TAC).

Experimental group	Mouse ID	Days post-surgery	Reason for death
WT sham	LAFW15.2b	21	Euthanized due to genital mutilation (unrelated to procedure)
OE sham	LAFW26.1d	1	Euthanized due to intra-thoracic bleed associated with surgery
WT-TAC	LAFW7.5b	1	Euthanized due to peripheral oedema
WT-TAC	LAFW15.3b	31	Died of congestive heart failure
WT-TAC	LAFW16.2b	1	Euthanized due to acute heart failure
WT-TAC	LAFW15.3f	1	Euthanized due to acute heart failure
WT-TAC	LAFW20.2e	6	Euthanized due to >15% weight loss (humane end-point)
WT-TAC	LAFW15.5a	2	Euthanized due to acute heart failure
WT-TAC	LAFW20.4b	3	Euthanized due to >15% weight loss (humane end-point)
WT-TAC	LAFW20.4d	6	Euthanized due to >15% weight loss (humane end-point)
WT-TAC	LAFW15.6c	8	Died from aortic aneurysm
OE-TAC	LAFW13.4c	11	Died of congestive heart failure
OE-TAC	LAFW18.5g	5	Euthanized due to >15% weight loss (humane end-point)
OE-TAC	LAFW26.2a	2	Euthanized due to acute heart failure

Supplementary Table 2 Causes of death for all mice not surviving until the 6 week haemodynamic examination.

	WT-SHAM	OE-SHAM	WT-TAC	OE-TAC
Baseline	*(n=10)*	*(n=12)*	*(n=14)*	*(n=19)*
Heart rate (bpm)	455 ± 17	448 ± 16	454 ± 10	487 ± 8
LV systolic pressure (mmHg)	102 ± 3	101 ± 2	149 ± 7 ***	127 ± 6 *
LV diastolic pressure (mmHg)	5.1 ± 0.7	3.7 ± 0.4	14.5 ± 2.7 *	17 ± 2.6 ***
dP/dt_{max} (mmHg/s)	8482 ± 521	8666 ± 495	6886 ± 441	5829 ± 483***
Tau (ms)	7.9 ± 0.4	7.4 ± 0.5	11.0 ± 1.1	12.2 ± 1.0
+ dobutamine	*(n=10)*	*(n=12)*	*(n=14)*	*(n=15)*
Heart rate (bpm)	544 ± 13	538 ± 10	518 ± 11	543 ± 8
LV systolic pressure (mmHg)	103 ± 3	101 ± 2	146 ± 7 **	137 ± 6 **
LV diastolic pressure (mmHg)	4.8 ± 0.5	3.8 ± 3.2	12.9 ± 2.5	14.3 ± 3.0 **
dP/dtmax (mmHg/s)	12444 ± 657	12870 ± 652	7714 ± 608***	7480 ± 736***
Tau (ms)	6.7 ± 0.3	6.3 ± 0.6	10.2 ± 1.2	9.8 ± 1.0 *

Supplementary Table 3 Left ventricular haemodynamic parameters measured 6 weeks after sham or TAC surgery. Data is mean ± standard error. * denotes $P<0.05$, ** $P<0.01$, *** $P<0.001$ for comparison between Sham and TAC groups within the same genotype. There were no significant differences between genotypes, i.e. between WT-Sham vs OE-Sham and WT-TAC vs OE-TAC.
